# Convolutional neural networks in the qualitative improvement of sweet potato roots

**DOI:** 10.1038/s41598-023-34375-6

**Published:** 2023-05-24

**Authors:** Ana Clara Gonçalves Fernandes, Nermy Ribeiro Valadares, Clóvis Henrique Oliveira Rodrigues, Rayane Aguiar Alves, Lis Lorena Melucio Guedes, André Luiz Mendes Athayde, Alcinei Mistico Azevedo

**Affiliations:** Universidade Federal de Minas Gerais, Instituto de Ciências Agrárias (ICA), Campus Regional de Montes Claros, Avenida Universitária, 1.000 – Bairro Universitário, Montes Claros, Minas Gerais 39.404-547 Brazil

**Keywords:** Plant sciences, Agricultural genetics

## Abstract

The objective was to verify whether convolutional neural networks can help sweet potato phenotyping for qualitative traits. We evaluated 16 families of sweet potato half-sibs in a randomized block design with four replications. We obtained the images at the plant level and used the ExpImage package of the R software to reduce the resolution and individualize one root per image. We grouped them according to their classifications regarding shape, peel color, and damage caused by insects. 600 roots of each class were destined for training the networks, while the rest was used to verify the quality of the fit. We used the python language on the Google Colab platform and the Keras library, considering the VGG-16, Inception-v3, ResNet-50, InceptionResNetV2, and EfficientNetB3 architectures. The InceptionResNetV2 architecture stood out with high accuracy in classifying individuals according to shape, insect damage, and peel color. Image analysis associated with deep learning may help develop applications used by rural producers and improve sweet potatoes, reducing subjectivity, labor, time, and financial resources in phenotyping.

## Introduction

Sweet potato (*Ipomoea batatas* (L.) LAM) is among the most consumed vegetables in Brazil because it is a rich source of carbohydrates, fiber, vitamins, minerals, and antioxidants and because of its low glycemic content^1^. In addition to multiple uses such as bioethanol production^2^, sweet potato is a vegetable that plays a relevant role in supplying raw materials for human and animal food^3,4^.

The main challenge for commercializing this vegetable is associated with its market value since it is strongly dependent on the qualitative characteristics of the roots, such as the shape^5^. Hence, consumers prefer products that have adequate commercial standards and good appearance. Thus, although deformed products have the same nutritional value as commercial roots, they are often discarded by consumers^5^, becoming a source of food waste.

It is necessary to invest in information and new technologies associated with the genetic improvement of vegetables for commercial purposes to increase products’ productivity and quality and minimize the losses of the rural producer^1^. However, in selecting the best genotypes, it is necessary to evaluate many quantitative and qualitative characteristics, which is an expensive and subjective process making the analysis difficult for the breeder^6^. In this sense, the adoption of new technologies associated with the phenotyping process represents an advance, and among the possibilities, we have image analysis connected to computational intelligence.

Using strategies that allow the acquisition and analysis of data from agricultural environments can help optimize current practices, promoting increased productivity, better quality control processes, and flexibility in agricultural management^7^. Moreover, these new technologies aimed to improve the accuracy and speed of phenotypic measurements have been the subject of intense research in recent years^8^, such as in the development of phenotyping platforms^9^, automated high-efficiency phenotyping systems^10^, and characterization of phenotypes in sweet potato using images^5^. We may use convolutional neural networks (CNNs) to automate the interpretation of these images.

Convolutional neural networks (CNNs) have become popular for object detection because they can classify objects and extract image descriptors^11^. Furthermore, they can achieve high performances for different classification and detection problems, achieving faster inference time and higher detection rates than traditional computer vision methods^12^. Thus, the association of images to convolutional neural networks has already been used for the automatic quantification of ears of wheat in the field^13^ and for the classification of the milling fraction of lentils and peas^14^.

In genetic improvement, the convolutional neural network has already been approached to predict phenotypes from genotypes^15^.Thus, using images associated with deep learning neural networks can be an alternative for efficiently classifying roots into commercial and non-commercial. Hence, the objective was to verify if convolutional neural networks can help phenotyping sweet potatoes for qualitative traits.

## Material and methods

### Declaration research involving plants

All methods were performed in accordance with relevant guidelines and regulations. The methodology used does not involve the use of human tests. The methodology was tested on images of sweet potato roots obtained by the authors themselves. Sweet potato is an easily propagated species and is not on the endangered species list.

### Installation and evaluation of the experiment

We carried out the experiment at UFMG Agrarian Sciences Institute—Montes Claros-MG Campus (ICA/UFMG) (coordinates: 16°40′58.16″ S and 43°50′20.15″ W) where 16 sweet potato families were evaluated in haplic cambisol under irrigation conditions [BELGARD (F2), CAMBRAIA (F4), LICURI (F5), UFVJM40 (F6), UFVJM01 (F7), ARRUBA (F8), UFVJM05 (F10), UFVJM15 (F13), UFVJM56 (F16), UFVJM31 (F20), UFVJM37 (F22), UFVJM54 (F24), UFVJM25 (F26), UFVJM29 (F27), TCARRO02 (F29), UFVJM09 (F25)].

We obtained the sweet potato half-sibs by collecting seeds from the germplasm bank comprised of elite accessions brought from the Federal University of the Jequitinhonha and Mucuri Valleys(UFVJM) and cultivated at ICA/UFMG. The seeds were collected daily between April and October 2018 and stored in a refrigerator at 4 °C. Subsequently, the seeds were subjected to mechanical scarification with sandpaper to break dormancy (tegumentary impermeability) and planted in 72-cell polystyrene trays with a commercial substrate. The trays were kept in a greenhouse and irrigated daily for two months when the seedlings were ready to be planted.

Planting was carried out in rows in a randomized blocks design (RBD) with 16 families (different progenies) and four replications, with rows spaced 1 m apart and spacing between plants of 0.4 m. Once we made the evaluations at the plant level, we used a larger spacing to facilitate identifying each plant and facilitate the harvest. We carried out fertilization and cultural treatments as recommended for the crop in the New Horticulture Manual^16^. We used 180 kg ha^−1^ of phosphorus and 30 kg ha^−1^ of nitrogen. Thirty days after planting the seedlings, we applied top dressing with 30 kg ha^−1^ of nitrogen. Potassium fertilization was not necessary according to the chemical analysis of the soil.

At first, we applied sprinkler irrigation every day to keep the soil with good moisture content. After the critical period of crop establishment (2 months after transplanting), we used irrigation twice a week.

Manual harvesting was performed 165 days after planting, carrying out analyses at the plant level when we removed excess soil from the roots to obtain images. We analyzed the variables shape, skin color, and damage caused by insects. The evaluations were carried out according to the descriptors and scoring scales recommended by the International Board for Plant Genetic Resources (IBPGR), elaborated by Ref.^17^ (Table 1).

**Table 1:** Score scale related to shape, predominant skin color, and damage caused by insects for half-sib progenies of sweet potato (Ipomoea batatas (L.) Lam).

Descriptor	Scores
Shape	1- round; 2- round elliptic; 3- elliptic; 4- ovate; 5- obovate; 6- oblong; 7- long oblong; 8-long elliptic; 9- irregular (Adapted)
Predominant skin color	1- white; 2- cream; 3- yellow; 4- orange; 5- brownish orange 6- pink; 7- red; 8- purple-red; 9- dark purple
Damage caused by insects	0- absent; 1- present

Source: Adapted from Ref.^17^.

Concerning shape, we classified the roots as commercial and non-commercial, with those with a more fusiform shape being considered commercial. Regarding skin color, we divided the roots into light-colored (white, cream, yellow, and orange) and dark-colored (pink, red, purple-red, and dark purple). As for the damage caused by insects, we classified the roots according to the presence and absence of damage.

### Image generation and processing

**Figure 1:**
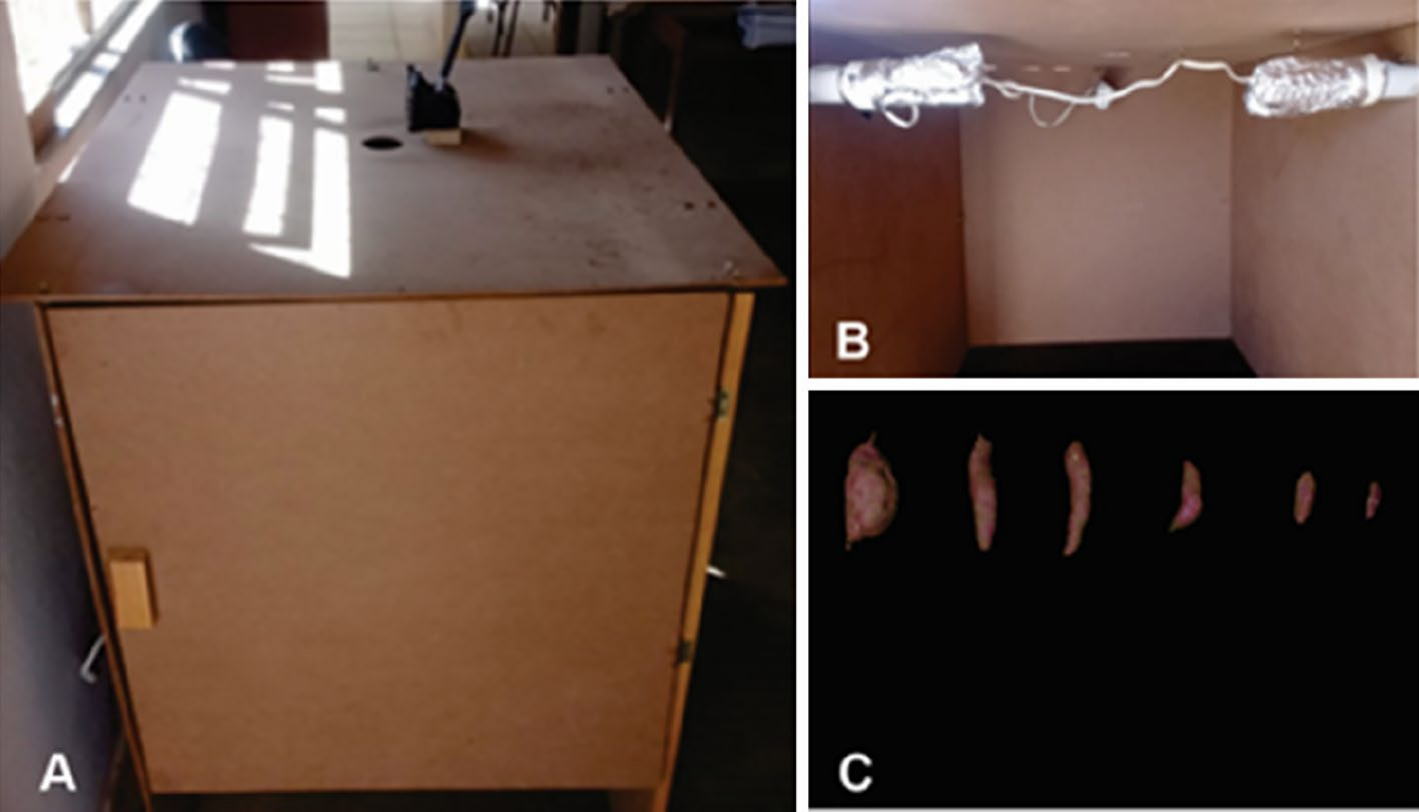
Image generation: (**A**) “Studio” for image generation; (**B**) Artificial lighting with a fluorescent lamp; (**C**) Image generated in the studio. Source: Authors (2022).

We generated images in a “studio” made of MDF (Medium Density Fiberboard) with dimensions of 0.50 × 1.00 m at the bottom and 1.0 m high (Fig. 1). We used a Canon PowerShotSX400 IS digital camera under artificial lighting with a fluorescent lamp. The camera was attached to a support to standardize image generation so that we could get all images from the same height (70 cm) and angle (90°). We placed the roots in front of a black background and spaced them apart without overlapping.

**Figure 2:**
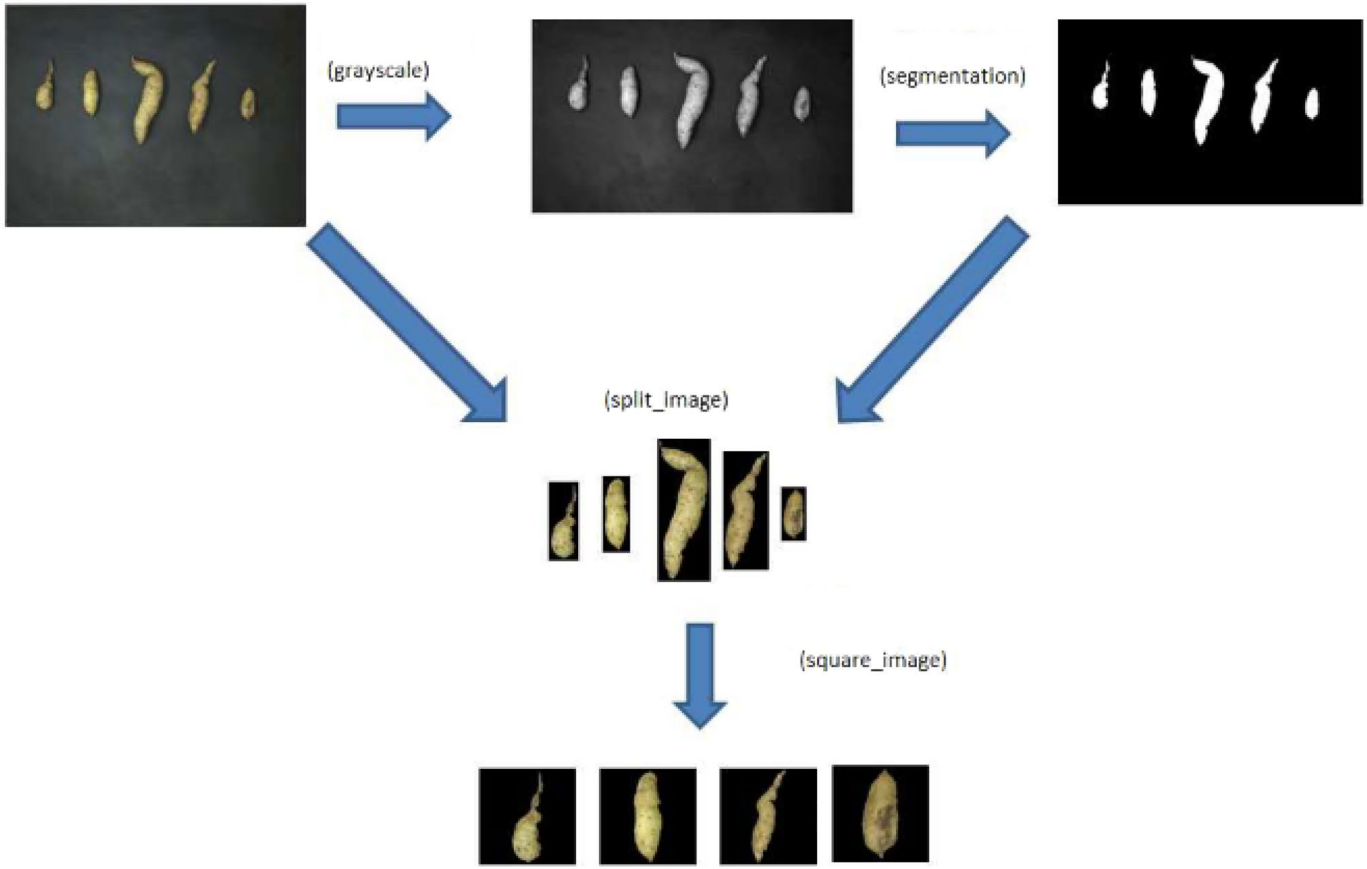
Steps for individualizing one root per image in the phenotyping of sweet potato roots by computational image analysis. Source: Authors (2022).

We used the ExpImage package of the R software to reduce the resolution and individualize one root per image (Fig. 2). We grouped these images according to their classifications regarding format (commercial and non-commercial), skin color (light and dark), and insect attack (damaged and undamaged).

We visually classified the images of sweet potato roots considering the variables of root skin color (light and dark), shape (commercial and non-commercial), and damage caused by insects (damaged and undamaged) (Table 2). We divided the original images for each variable into the ones destined for adjustment of recurrent correlation neural networks (RCNNs) and to evaluate goodness of fit (Test). Thus, we set 600 roots of each classification to train the networks, while the remainder was used to verify the goodness of the fit (Table 2).

**Table 2:** Number of images of sweet potato roots used for phenotyping color, shape, and insect damage (originals) and the number of images destined for the adjustment of RCNNs (Trainig) and evaluation of the goodness of fit (Test).

Classes	Originals	Training	Test
Color			
Light	1160	600	560
Dark	1198	600	598
Shape			
Commercial	1244	600	644
Non-commercial	744	600	144
Insect attack			
Damaged	744	600	144
Undamaged	1614	600	1014

Source: Authors (2022).

We replicated each of the images with four different rotations (45°, 135°, 225°, and 315°) to expand the training dataset. Thus, for each classification, there were 3,000 images in training (600 + 4 × 600). We used the python language on the Google Colab platform for training the networks and the Keras library, considering the VGG-16, Inception-v3, ResNet-50, InceptionResNetV2, and EfficientNetB3 architectures. We considered the maximum of 100 iterations and early stopping with a tolerance of 5 iterations.

We constructed confusion matrices to evaluate the adjustments of the convolutional networks, with the classifications predicted by the different network architectures as a function of the visual classifications. We used the metrics Recall (Eq. (1)), Accuracy (Eq. (2)), Precision (Eq. (3)), F-Measure (Eq. (4)), and Specificity (Eq. (5)) to assess the network efficiency^18^. Where: TP refers to true positives, FN to false negatives, FP to false positives, and TN to true negatives.

1 $$Recall= \frac{TP}{TP+FN}$$

2 $$Accuracy= \frac{TP+TN}{TP+TN+FP+ TN}$$

3 $$Precision= \frac{TP}{TP+FP}$$

4 $$F-measure= \frac{2*Precision*Recall}{Precision+Recall}$$

5 $$Specificity= \frac{TN}{FP+TN}$$

## Results

For each architecture, it was possible to observe different epochs required for training (Table 3). For shape, damage caused by insects, and skin color, the architectures Inception-v3 and InceptionResNetV2 presented the lowest classification times and the lowest number of epochs (Table 3). However, for all analyzed variables, there was a higher rate of true positive (TP) ratings for the InceptionResNetV2 architecture. On the other hand, the EfficientNetB3 architecture was the one that presented the lowest efficiency for detecting shape and damage caused by insects in the sweet potato roots, demanding a higher number of epochs and, consequently, a longer time for classification (Table 3). While for skin color, the ResNet-50 architecture showed lower efficiency.

**Table 3:** Number of epochs, training time, and classifications performed by different architectures of RCNNs for sweet potato roots regarding shape, damage caused by insects, and skin color. UFMG (2022).

Var	Architecture	Epochs	Time	TP	FN	FP	TN
Shape	VGG-16	70	0:29:58.5	517	127	132	382
	Inception-v3	25	0:12:00.3	543	101	158	356
	ResNet-50	76	0:31:48.1	526	118	160	354
	InceptionResNetV2	17	0:17:59.3	630	14	20	494
	EfficientNetB3	100	1:05:30.8	567	77	138	376
Damage caused by insects	VGG-16	95	0:36:46.8	97	46	299	715
	Inception-v3	18	0:07:47.7	111	32	99	915
	ResNet-50	24	0:28:16.3	73	70	251	763
	InceptionResNetV2	11	0:06:27.7	140	3	38	976
	EfficientNetB3	100	0:45:22.6	87	56	262	752
Skin color	VGG-16	80	0:34:56.5	464	96	70	528
	Inception-v3	31	0:15:07.8	455	105	184	414
	ResNet-50	98	1:20:17.9	481	79	98	500
	InceptionResNetV2	32	0:34:53.3	540	20	2	596
	EfficientNetB3	98	1:06:43.3	505	55	54	544

*TP* true positives, *FN* false negatives, *FP* false positives, *TN* true negatives.

Source: Authors (2022).

The efficacy of the convolutional neural network architectures in classifying the sweet potato roots according to the shape considered ideal, the damage caused by insects, and the root skin color, was evaluated using scores of precision, recall, F-measure (F1), accuracy, and specificity (Table 4). The InceptionResNetV2 architecture was the one that obtained the best precision, accuracy, and specificity for all analyzed variables. This architecture enabled accuracy close to 78.7% to classify damage caused by insects. This metric was superior to the accuracy obtained by other architectures for the same variable. Concerning the other architectures, all evaluation metrics were below 91% for the three analyzed variables.

**Table 4:** Evaluators of the goodness of fit for RCNNS with different architectures in classifying sweet potato roots according to shape, damage caused by insects, and skin color. UFMG (2022).

Var	Architecture	Precision	Recall	F1	Accuracy	Specificity
Shape	VGG-16	0.797	0.803	0.800	0.776	0.743
	Inception-v3	0.775	0.843	0.807	0.776	0.693
	ResNet-50	0.767	0.817	0.791	0.760	0.689
	InceptionResNetV2	0.969	0.978	0.974	0.971	0.961
	EfficientNetB3	0.804	0.880	0.841	0.814	0.732
Damage by insects	VGG-16	0.245	0.678	0.360	0.702	0.705
	Inception-v3	0.529	0.776	0.629	0.887	0.902
	ResNet-50	0.225	0.510	0.313	0.723	0.752
	InceptionResNetV2	0.787	0.979	0.872	0.965	0.963
	EfficientNetB3	0.249	0.608	0.354	0.725	0.742
Skin color	VGG-16	0.869	0.829	0.848	0.857	0.883
	Inception-v3	0.712	0.813	0.759	0.750	0.692
	ResNet-50	0.831	0.859	0.845	0.847	0.836
	InceptionResNetV2	0.996	0.964	0.980	0.981	0.997
	EfficientNetB3	0.903	0.902	0.903	0.906	0.910

Source: Authors (2022).

**Figure 3:**
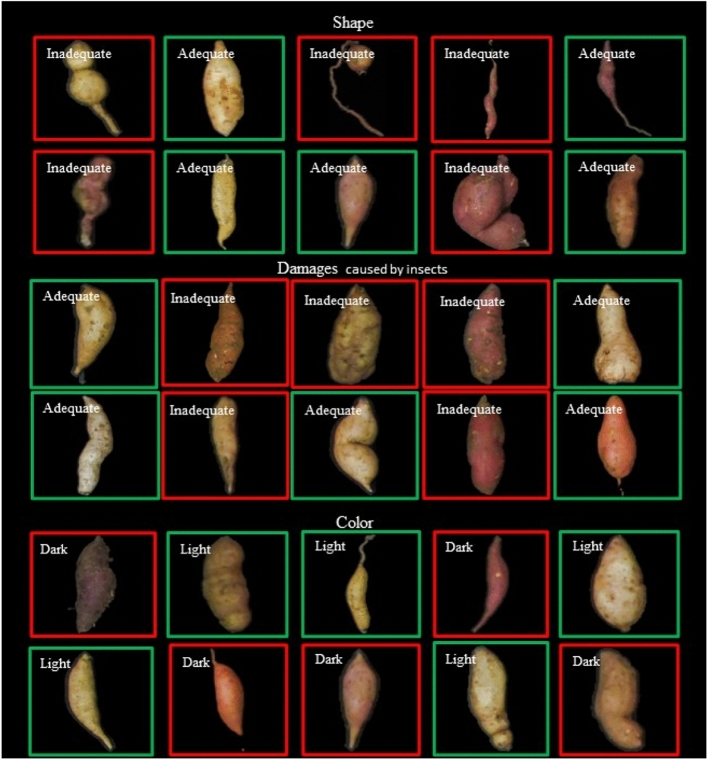
Classification of randomly selected roots in the samples from test images by RCNN InceptionResNetV2 for classifying sweet potato roots in terms of shape, damage caused by insects, and skin color. UFMG (2022). Source: Authors (2022).

In Fig. 3, to visualize the classification of roots randomly selected from the test samples by InceptionResNetV2, it was possible to note the accuracy of the network in classifying commercial individuals in terms of shape, damage caused by insects, and skin color. Inadequate individuals (red boxes) are those with an irregular shape, damaged by insects, and dark in color.

Thus, for the studied population, classification and, consequently, phenotyping of sweet potato roots in terms of shape, damage caused by insects, and skin color, can be performed efficiently and in a shorter response time with the architecture InceptionResNetV2. However, it is worth mentioning that only two classes (adequate and inadequate) are relevant for the initial phase of genetic improvement. We must detail these classes in later improvement steps.

## Discussion

Convolutional neural networks (CNNs) are a trend in image information processing due to their adaptability and efficiency in object detection^7^. The learning process of networks occurs through an iterative process of adjustments applied to the synaptic weights (training) when the neural network reaches a generalized solution for a given problem^19^. However, the greater the number of iterations used in training, the greater the memorization of data by the networks tends to be, resulting in the non-general character of the system (overfitting). Thus, the fitting process, the heavy computational load, the high tendency of overfitting and the empirical nature of model establishment are the main limitations associated with deep CNNs^20^. Thus, defining an optimal number of iterations for the analyzed datasets is essential. That can be done by using a strategy called early stopping. For each type of architecture and for each variable used, it was possible to observe different epochs, where the Inception-v3 and InceptionResNetV2 architectures presented the lowest number of epochs and, consequently, the lowest classification times (Table 3). That indicates the reliability and efficiency of the method, in addition to saving time in obtaining results^21^. On the other hand, the EfficientNetB3 and ResNet-50 architectures were the ones that presented the lowest efficiency in the classification of the studied dataset and also had the highest number of epochs and the longest time to obtain the results.

Selecting the architecture that enables analysis more efficiently allows us to qualify features in large datasets with little labor force. That can help breeders evaluate the interaction genotype x environment more effectively, leading to the identification of potential new cultivars in a shorter period of time^5^. Thus, the higher the true positive rate identified by each architecture for the variables root shape, damage caused by insects, and skin color, the greater the precision and, consequently, the recall, accuracy, and F-Meansure. That is corroborated in the present study by the InceptionResNetV2 architecture since we obtained better precision, accuracy, and specificity for all analyzed variables, with the precision for this network higher than 91% for the variable shape and skin color and greater than 78% for damage caused by insects (Table 4). By using sweet potato images to train a neural network classifier for sweet potato root shape, Ref.^5^ obtained lower precision than those in the present study. This difference in accuracy may be associated with the classes used by each researcher to consider an ideally-shaped root. This high accuracy of the architectures used in root classification can further improve the decision-making process in agricultural practices^7^.

The different responses obtained by the tested architectures are directly associated with the construction of CNN's, and may vary according to size, precision, number of parameters, depth and time per inference^22^. In this case, the InceptionResNetV2 architecture benefits from the other tested architectures due to the integration of two well-known deep convnets, Inception and ResNet, which contribute positively to the more accurate result in this study. This also suggests that features extracted from different convnets are complementary and improve the model classification efficiency^23^. The results showed that deeper networks (e.g. InceptionResNetV2) are more efficient in separating the input space into more detailed regions, due to its deeper architecture, which contributes to a better detection of the studied classes.

We saw in Fig. 3 the efficacy of classifying sweet potato roots by computational analysis using the InceptionResNetV2 architecture. For this purpose, bounding boxes were applied, which allowed a demonstration of data categorization. Through this approach, the neural network can classify sweet potato roots for each analyzed characteristic. The high efficiency obtained by the developed system can also be justified by the absence of overlapping objects, allowing greater accuracy in identification^24^. In addition, another factor that may have influenced the metrics is the sharpness of root coloring since defective areas may have a different color pattern from the rest of the root, improving classification accuracy and providing new information about root quality^5^. Thus, the easier the distinction of the image RGB matrix in terms of its objects and parts, the greater the chance of success in detecting the evaluated classes^25^.

We can infer from the results that the developed system efficiently classifies sweet potato roots, making the interpretation process faster, more accurate, and less subjective. That is one of the main differentials of the technique developed since most of the research investigating size and shape characteristics of horticultural crops is carried out on a laboratory scale, not adapted to large-scale production^26,27^. Therefore, the developed system has great potential to be adapted and used to collect and analyze data by small-scale producers and on large commercial scales. That helps not only in the phenotyping process of the crop but also in separating roots considered commercial or not. For genetic improvement, the quantification of roots belonging to each class may be used as a selection criterion, either individually or simultaneously, adding relevant information for better genetic progress of the crop.

Thus, this approach makes it possible to quantify the loss due to shape deformation and damage caused by insects. However, a prime challenge in implementing this method is the computational power required to process hundreds of thousands of roots, which requires expensive computers. One way to mitigate this challenge is to use cloud servers^5^. In addition, the developed methodology opens the way to investigate other horticultural crops, indicating the possibility of developing treadmill equipment with cameras for high-scale phenotyping, either for commercial purposes or for genetic improvement.

## Conclusions

The InceptionResNetV2 architecture performed better in classifying individuals according to shape, damage caused by insects, and skin color, obtaining high estimates for the parameters used to assess the goodness of fit.

Image analysis associated with deep learning may improve the quality of sweet potato roots, reducing analysis subjectivity and the time of phenotyping the culture.

We believe that the efficiency of the methodologies used can provide valuable information and tools to researchers, substantially contributing to the future development of applications and devices for the classification of sweet potato roots, in order to help rural producers, traders and breeders. However, studies must be deepened in order to incorporate libraries or cloud servers for root classification.

## Data Availability

The data that support the findings of this study will be made available by the authors upon prior request. Data can be requested from the authors Ana Clara Gonçalves Fernandes (anaclaragoncalvesfernandes@gmail.com) and Alcinei Mistico Azevedo (alcineimistico@hotmail.com).
